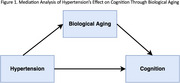# The Role of Biological Aging in Mediating the Association Between Hypertension and Cognitive Function in Multi‐ethnic Elderly Cohort

**DOI:** 10.1002/alz70860_107624

**Published:** 2025-12-23

**Authors:** Tristin Yun, Anisha Das, Jenny J Lee, Annie J. Lee

**Affiliations:** ^1^ Tenafly High School, Tenafly, NJ, USA; ^2^ Columbia University Department of Neurology, New York, NY, USA; ^3^ Ewha Womans University, Seoul, Seoul, Korea, Republic of (South); ^4^ Department of Neurology, Columbia University Medical Center, New York, NY, USA; ^5^ Taub Institute for Research on Alzheimer's Disease and The Aging Brain, Columbia University Medical Center, New York, NY, USA; ^6^ The Gertrude H. Sergievsky Center, College of Physicians and Surgeons, Columbia University, New York, NY, USA

## Abstract

**Background:**

Vascular risk factors, including hypertension, are associated with cognitive decline. Hypertension is also associated to the maintenance of metabolic, immune, and physical functions during aging. These observations suggest that hypertension may contribute to accelerated biological aging. We aim to investigate whether biological aging mediates the effect of hypertension on cognitive function.

**Method:**

We analyzed data from the National Health and Nutrition Examination Survey (NHANES) 2011–2014. Hypertension was defined using self‐reports, direct measurements, or antihypertensive medication use. Global cognitive function was assessed using a composite z‐score based on CERAD Word Learning and Recall, Animal Fluency, and Digit Symbol Substitution tests. Biological aging was measured using nine blood biomarkers (albumin, alkaline phosphatase, C‐reactive protein, creatinine, glucose, white‐blood‐cell count, lymphocyte %, mean cell volume, and red‐cell distribution width). Using causal mediation analysis (Figure 1), we investigated whether biological aging mediates the association between hypertension and cognitive function, adjusted for sex, education, body mass index, annual income, and race/ethnicity. We tested heterogeneity in these associations across different race/ethnicity.

**Result:**

The study included 2,421 adults aged 60‐80 years (51% women, 62.1% with hypertension, mean age 69.1, 23.6% with accelerated biological aging, i.e., biological age > chronological age). Hypertension was associated with older biological age (*b* = 3.34, *p* < 0.001). Biological aging mediated 41% of the effect of hypertension on cognitive function (mediation effect *b* = ‐1.59, 95% CI: ‐2.02 to ‐1.18, *p* < 0.001). The mediation effect of biological aging was particularly pronounced in Mexican Americans, where it accounted for 26% of the hypertension‐cognition association, while no significant mediation was observed in non‐Hispanic White or non‐Hispanic Black participants.

**Conclusion:**

Among a diverse sample of older adults, biological aging, as measured by biomarkers, was found to mediate the relationship between hypertension and cognitive function. This mediation effect varied across racial/ethnic groups, highlighting the need for tailored interventions to address race/ethnic differences and mitigate hypertension‐related cognitive decline.